# Parental perception of neonatal transfers from level 3 to level 2 neonatal intensive care units in Calgary, Alberta: qualitative findings

**DOI:** 10.1186/s12913-021-06967-3

**Published:** 2021-09-17

**Authors:** Aliyah Dosani, Prashanth Murthy, Shafana Kassam, Baldeep Rai, Abhay K. Lodha

**Affiliations:** 1 School of Nursing and Midwifery, Mount Royal University, Calgary, Canada; 2 Department of Community Health Sciences, University of Calgary, Calgary, Canada; 3 O’Brien Institute for Public Health, University of Calgary, Calgary, Canada; 4 Cumming School of Medicine, University of Calgary, Y458, 4825 Mount Royal Gate S.W., Calgary, AB T3E 6K6 Canada; 5 Department of Pediatrics, University of Calgary, Calgary, Canada; 6 Alberta Health Services, Calgary, Canada; 7 Faculty of Arts and Science, University of Toronto, Toronto, Canada; 8 Alberta Children’s Hospital Research Institute, University of Calgary, Calgary, Canada

**Keywords:** Parental perceptions, Retro-transfer, Level 3 NICUs, Level 2 NICUs

## Abstract

**Background:**

Retro-transfers from level 3 to 2 NICUs in Alberta’s regionalization of neonatal care system are essential to ensure the proper utilization of level 3 NICUs for complex neonatal cases. Parents often experience distress that relates to the transfer of their neonates to another hospital. Limited information is available regarding parental perceptions of distress during transfers for neonates requiring care between NICUs in the current Canadian context. The objective of this study was to investigate: 1) what caused parents distress and could be changed about the transfer process and 2) the supports that were available to help ease parental distress during the transfer process.

**Methods:**

Parents of singleton infants retro-transferred from level 3 to 2 NICUs in Calgary, Alberta between January 1, 2016, and December 31, 2017, were invited to participate in the study. Questionnaires were self-administered by one parent per family. A thematic deductive approach was employed by the researchers to analyze the qualitative data.

**Results:**

Our response rate was 39.1% (*n* = 140). We found three themes for causes of parental distress and supports available to ease parental distress during the transfer, including communication between staff members and parents, details about the transfer process, and the care received throughout and shortly after the transfer process.

**Conclusion:**

Parents should receive at least 24 h of notice, regular transfer updates, employ anticipatory preparation strategies, and foster more open communication between parents and health care professionals to help ensure parental satisfaction.

## Introduction

The preterm birth rate in Alberta was 8.7%, the second highest among the Canadian provinces [1]. Alberta follows a regionalization system of neonatal intensive care units (NICUs) based on the level of care they provide [2]. The term “retro-transfer” is used to describe the transfer of a neonate from a higher to a lower level of care once they become more stable [3, 4]. Retro-transfers ensure appropriate utilization of level 3 NICU beds, resources, and health care professionals’ skills when caring for infants with complex cases [2]. Southern Alberta has an annual birth rate of approximately 24,000 and only 54 level 3 NICU beds available. Therefore, retro-transfers are essential to maintain continued access to the appropriate level of care.

However, the retro-transfer process is not without challenges. Parents often experience distress related to their neonate’s transfer to another hospital [5, 6]. The distress experienced can be explained in terms of physical separation, perceived changes in the parenting role and disruption of parenthood, and feelings of uncertainty [7–9]. Parents feel anxious and worry about their neonate’s health during the physical transfer process, the competency of the new health care team, and being in an unfamiliar physical setting [10]. Furthermore, parents felt like the transfer process undermined their role as a nurturer [10, 11], were upset about not being involved in the transfer decision [11, 12], and the physical and emotional separation during the transfer threatened parents’ roles as caregivers [9, 11, 12]. Lastly, receiving inadequate information to prepare for the transfer, including not being part of the decision-making process and perceived differences in cultures of care between facilities, were identified as significant sources of stress for parents [9, 11, 12]. There is a paucity of research investigating parental perceptions of distress during transfers for neonates between NICUs in the current Canadian context [5, 9]. Therefore, using a deductive approach [13], we wished to explore if the factors causing distress in other countries were similar in present-day Canada. The objectives of this study were to investigate the following research questions regarding retro-transfers from level 3 to level 2 NICUs in Calgary: 1) What caused parents distress and could be changed about the transfer process? and 2) What supports were available to help ease parental distress during the transfer process?

## Methods

We used a survey design to collect and analyze quantitative and qualitative data [14]. We developed a questionnaire, with quantitative and open-ended written questions, self-administered by one parent per family. The qualitative portion of our survey was informed by the phenomenological method as this research explores the nature of an experience from the people experiencing the phenomenon, in this case, retro-transfer [15, 16]. Guided by phenomenological approaches, we framed questions in a way to elicit in-depth responses about parental experiences of their infant’s retro-transfer. Taking a phenomenological approach to developing the questionnaire helped us understand parents’ lived experiences on a deeper level by exposing what health care professionals were taking for granted in terms of their assumptions about the transfer process [17]. Face validity was established by pilot-testing the questionnaire with three families to ensure that the questions accurately measured the research questions and that families understood what was being asked. Additionally, content validity was confirmed by reviewing the pre-existing literature to ensure that all aspects of the transfer experience for parents of neonates requiring intensive care were considered.

**Fig. 1:**
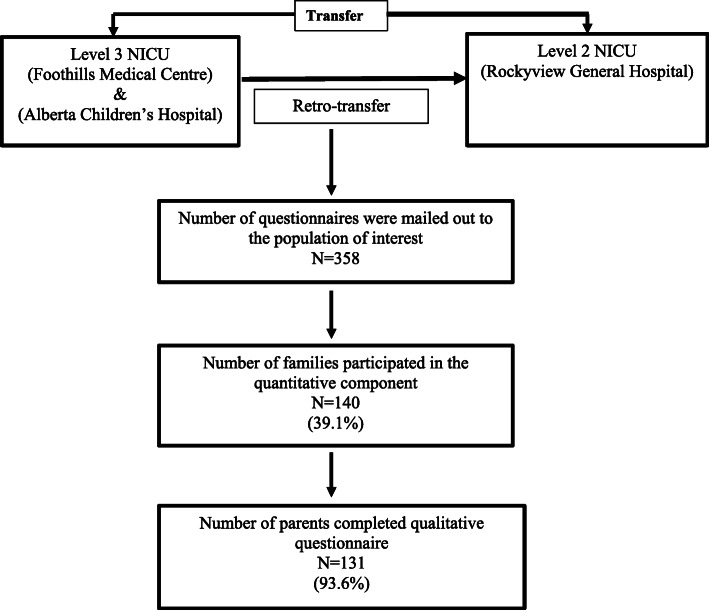
Flow diagram of the recruitment process

All parents of singleton neonates transferred from either of the two-level 3 NICUs to a level 2 NICU in Calgary, Alberta, between January 1, 2016 and December 31, 2017, were invited to participate in the study. Questionnaires were mailed out to the population of interest following their infant’s discharge from the level 2 NICU. Details about the recruitment process are shown in Fig. 1. Additionally, two rounds of telephone calls reminding parents about the study were made in April and June 2018. During this two-year period, there were no changes to procedures or guidelines relating to retro-transfers in any of the NICUs participating in our study. We analyzed the qualitative data using a thematic deductive approach, which is used to test a previous theory in a different context or to compare categories at different time periods [13, 18, 19].

We used an interpretive thematic analytic method, an iterative and inductive process involving decontextualization and recontextualization [17], and integration which involved inductive and deductive reasoning. Researchers decontextualized the information to significant statements or quotes, coded the statements into themes, and combined statements of textural and structural descriptions to convey the essence of the experience [17, 20, 21]. The researchers then identified patterns in the codes and distinguished and acknowledged central themes and relationships across participants and narratives [17]. This process is referred to as recontextualization, where researchers will display data and permit the identification of emerging patterns and interrelationships and assist in drawing conclusions in a new way to answer the research questions [22]. We drew conclusions where the themes were both valid and sound. A theme was valid if it was impossible for the premises underlying the themes to be true while its conclusion was false. A theme was sound if it was valid, and the premises underlying the themes were true. It is in this way that we incorporated deductive reasoning. Furthermore, phenomenological approaches include interpreting the structures of experiences and how things are understood by people who live through these experiences [16, 23]. Upon completing our thematic analysis, we came to conclusions to answer the research questions [19]. Ethics approval for this study was obtained from the University of Calgary’s Conjoint Health Research Ethics Board [Ethics ID: REB18-0240].

## Results

Quantitative results of our study are published elsewhere [24]. Demographic information about the study population is shown in Table 1. The final sample is representative of the high-income population that the level 2 NICU serves. We identified three themes from each research question from our qualitative data.

**Table 1:** Socio-demographic characteristics of participants (*N* = 140)

Variables	N (%)
Respondents	
Mother	125 (91.24)
Father	12 (8.76)
Current Relationship Status	
Non-married	7 (5.07)
Married	131 (94.93)
Postal Code	
Calgary	111 (82.22)
Other AB/SK/BC	24 (17.78)
Education	
High school and College	42 (31.11)
Undergraduate, Masters, PhD	93 (68.89)
Income	
< 20,000 to < 60,000	25 (19.08)
≥ 61,000	106 (80.92)
Were you born in Canada?	
Yes	92 (66.67)
No	46 (33.33)
Ethnicity	
White	96 (69.57)
Other Ethnicities	42 (30.43)
Language	
English	114 (82.61)
Others	24 (17.39)

### Question 1: what caused you distress and could be changed about the transfer process?

In response to the first question, parents spoke about what caused them distress and could be changed about the transfer process. Three major themes were found in the analyses of the interviews: the need for increased communication between the NICU staff and parents, the retro-transfer process itself, and care received during and shortly after the transfer process.

*Need for increased communication between the staff and parents: “It was sprung on us quickly and felt like we had no say in it. They were just taking our baby.”*

Parents commented on the lack of communication with them at both level 3 and level 2 NICUs. Participant #95 explained how “*more communication in a timely manner regarding when our son would be moved*” would have been beneficial. Participant #107 wanted to “*be informed*” and “*not get to [the level 3 NICU] and be told that my baby is gone!*” Increased communication would have permitted parents to plan for the transfer ahead of time since some lived “*somewhere else in the city*” (participant #40) and had to travel a greater distance to reach the level 2 NICU compared to the level 3 NICU.

*More notice regarding the transfer: “The short notice didn’t give me time to process what was happening and ask all questions.”*

The amount of notice given before the transfer represented the most significant issue for some parents. “*Advance notice that the transfer may occur and what the process would be*” (Participant #69) and “*adequate time to prepare for the transfer*” (Participant #81) were recurring concerns. Participant #37 specifically requested *“24-48 hours of notice regarding the transfer of the infant”. “On the day of actual transfer there wasn’t much warning if it was actually happening”* (Participant #84)*.* While participant #51 noted that although advanced notice was preferable, “*we understand this is operationally not always possible.*”

*More information regarding the transition between level 3 and level 2 NICUs: “education around how a level 2 NICU functions.”*

Due to the emotional and stressful nature of the transfer process, parents were concerned about having insufficient information regarding their transition from level 3 to level 2 NICUs. “*It was a culture shock going into [level 2] after [level 3]*” (Participant #53). First-time encounters with health care providers contributed to parents’ anxiety during this process. Participant #36 expressed feeling “*uneasy when watching nurses attend to alarms.*” Overall, parents “*would have liked to know more about the difference between [level 3] and [level 2] NICUs …*” (Participant #14) before being transferred.

*The risks associated with the transfer process:* “*… baby may struggle …”*

Some parents were unaware of the risks related to the transfer. For example, participant #14 “*[would] have [liked to] been told that [their] babies would [find] the transfer taxing*”. Many parents were unaware of the consequences it would have on their baby’s well-being, resulting in shock. For instance, participant 129 indicated, “*It would have been nice to know he would have a hard time with the transfer and may regress.*” Therefore, it is essential for health care providers to inform parents of the potential risks to the neonate’s health before the physical transfer process.

*Details about the transfer process itself: “I … would have liked to have been given the opportunity to ride the ambulance with my baby.”*

Being able to accompany one’s baby while being transferred was a concern for some parents. For instance, participant #6 explained how “*it would have been nice to go in the ambulance during the transfer or at least follow the ambulance.*” Participant #59 would have liked to stay connected via a “*webcam on the journey.*” Besides, some mothers were still patients at the facility that housed the level 3 NICU while their infant was getting transferred to the level 2 NICU. Participant #3 explains “*I wish I had also been transferred and my baby and I could continue to receive care jointly as one patient unit, instead of being [separated] when it was so new.*” Therefore, there is a need to identify various potential options for parents to be either transferred together or accompany the infant during the transfer process.

*More information during the transfer: “More alerts about where baby was during transfer.”*

Some parents expressed concerns about having inadequate information about the physical transfer. During the transfer, participant #40 “*found it stressful to not know where she was, who she was with or how the transfer went until later that day.*” Involving parents in the process and providing them with all the information they may need would reassure them about the transfer. Also, some parents would have liked more specific details regarding the transfer. Participant #115 indicated “*this was one of the hardest days we have had to endure, separated from my baby. I was terrified about the traffic. I wanted to know what route they were taking and who was meeting her … I got few real answers.”*

*Care received throughout and shortly after the transfer process: “Both my wife and I felt that there were inconsistencies in care.”*

After the transfer process, “*easing parents into the different style of care*” (participant #47) would have decreased parental anxiety. The transition from a level 3 to a level 2 NICU can be overwhelming. The level of care received in terms of healthcare personnel’s response times differ between the two levels, can be a significant adjustment. Participant #45 stated that “*more information on the practices and procedures at the new hospital*” was required because “*we were just kind of put there and it was a very different environment. It was a lot more relaxed than where we were coming from.*” Some encounters post-transfer with healthcare staff contributed to parental stress. “*Our son regressed on feeding for 3 days. [It] really bothered me and I had a nurse tell me that I cry too much … that was a very inappropriate response and could have hurt my mental health more*” (Participant #23). Several mothers did not feel supported *“[The] RN criticized how [I] was pumping, although I had been pumping for almost 100 days and knew what worked for me.* (Participant #12). Furthermore, “*nurses sometimes gave us conflicting information that was difficult. It [was] mostly about how much we could hold the babies and when [I] was allowed to bring them to my breast*” (Participant #5). Ensuring parents are given accurate and consistent information regarding their baby’s status and needs is crucial during this challenging time. Participant #3 expressed not feeling welcomed at the level 2 NICU: “*During the 2 weeks I was there, I was often told to go home and take care of myself first. Being treated as a disruption in my baby’s care was super-demoralizing and being [separated] from her [was] awful.*” Participant #37 expressed a similar concern and explained how “*we were not always made to feel welcome as parents spending extended periods of time in [the level 2] NICU.”*

Some parents indicated that some nurses were making errors with respect to feeding. The nurses “*seemed more rushed and overconfident while making mistakes such as incorrect feed volumes, missed feeds (over 3 hours late)*” (Participant #47). Similarly, “*one of [the] nurses set his feeding rate too low for 2 days. We as parents noticed that the calculation was not done correctly and then it was corrected. This was an unacceptable error.”* (Participant #71). To be a part of the care process as much as possible may have reduced parental anxiety. “*[We] would have loved more support of mom and dad doing as much care as possible (i.e. letting dad take temperatures/diaper changes) we had been doing this for 2 weeks prior to our [transfer] and when we arrived at [the level 2 NICU], it is like our child was no longer ours.*” (Participant #129).

### Question 2: what supports were available to help ease parental distress during the transfer process?

In response to the second question, parents spoke about what eased their distress during the transfer process. Our analysis revealed three major themes similar to those of the first research question: sufficient communication between the NICU staff and parents, the retro-transfer process itself, and care received throughout and shortly after the transfer process.

*Sufficient communication between parents and staff - “… open communication about the process …”*

Parents commented on the level of communication and information received from the staff at both level 3 and level 2 NICUs. Some parents believed that “*both [level 2 and level 3 staff] were very calm and informative about the process*” (Participant #102). Upon arrival at the level 2 NICU, “*the nurse welcomed and [gave] me some sort of orientation about [the] unit in general and some expectations.* (Participant #83). Being able to understand that the transfer process is a significant change for parents is important when building the initial relationship with parents.

*Reassurance about the transfer process: “I knew he was in good hands.”*

Due to the emotional and stressful nature of the transfer process, parents were satisfied with the reassurance they received from the healthcare staff at the two hospitals. “*Our nurse described in minute detail all aspects of the transfer process. Who goes in the ambulance, lights, sirens - off, what they track, what happens if there is a problem, etc.*” (Participant #18). Being provided with information about the process itself reassured parents that their baby would be transferred safely. Similarly, participant #36 expressed being reassured consistently by nurses “*about how skilled the transfer team was.”*

*Details about the transfer process itself: “It was very efficient.”*

The transfer process itself reassured parents that they were “*one step closer to home*” (Participant #27). “*Moving to [level 2 NICU] meant that she was progressing rather than regressing*” (Participant #77). Participant #60 was “*shocked by the amount of people, resources that appeared to move our baby girl safely and efficiently. It was amazing!”* Participant #69 explained that *“There was no ‘missing’ time and the transfer itself was quick.*” Overall, some parents were satisfied with the efficiency of the transfer process and the cooperation of the nurses and ambulance staff.

*Care received throughout and shortly after the transfer process: “… family centered care …”.*

Some parents were satisfied with the care they received throughout the transfer process. Some were familiar with the nurse that was accompanying their baby to the level 2 NICU. “*The nurses from [level 3 NICU] … accompanied our babies and waited for us … They left after confirming that babies and parents were fine*” (Participant #9). Participant #41 was pleased that “*the nurses at [level 2 NICU] wrapped my babies in heated blankets and tube fed them to give them rest. I loved that*”. Many parents were assured that this was a step in the right direction.

## Discussion

Three similar themes emerged from our results for both research questions. This affirmed that the factors which caused parental distress could be corrected and serve to ease the distress that parents experienced during the transfer process. These themes were communication between staff members and parents, details about the transfer process, and the care received throughout and shortly after the transfer process. With respect to communication between parents and staff members, parents would have liked to receive at least 24 h’ notice about the transfer. Many parents were unaware that the transfer would be occurring. We offer that having a formalized process for regular communication would ensure parents were up to date on their neonate’s care plans. When parents had regular interactions with a single health care team, parents felt they were more engaged in decision-making [9, 25, 26]. Our results are similar to Ballantyne et al. [5], who found that when increased engagement, open communication in the form of information sharing, and shared decision-making between health care providers and parents help enrich parents’ early transition experiences. Open communication lines and shared decision-making are essential to ensure no miscommunication may increase parental distress during an already difficult time.

Parents in our study would have liked to receive communication regarding the differences between the levels of care provided at the two different facilities. Hanrahan et al. [26] suggest that having the opportunity to visit the new facility before the transfer may acclimatize parents to the new care environment. We agree that anticipatory preparation and a formal orientation to the new facility would alleviate the “transition shock” some of the parents in our study experienced [3]. Future studies should focus on various interventions used to reduce parental distress before, during, and after the neonatal transfer processes.

Our research has a few limitations that impact the generalizability of our findings. Firstly, our results may have benefitted from employing individual interviews, rather than open-ended questions for our qualitative component. Using individual interviews would have resulted in more in-depth lived experience data as we would have had the opportunity to ask various probing questions according to the phenomenological method [16]. However, we did not have the resources required to complete interviews. Additionally, this is balanced by the broader number of participants that we were able to reach using the surveys. While we acknowledge this limitation, we were able to hold true to various other features of phenomenological research methods, including philosophical underpinnings, our approach to formulating questions, sampling criteria, analytical methods, target audiences and results obtained, as described by Starks and Trinidad [17]. Secondly, collecting data retrospectively introduces attrition bias and data degradation and social desirability bias [27]. One way to overcome this bias would have been to conduct a thorough chart review to ensure that the data we collected aligned with that of the qualitative notes of health care providers. Unfortunately, conducting chart reviews was outside of our scope. Thirdly, our study spanned 2 years that might introduce a recall bias. Fourthly, our response rate was low (39%) and even lower for fathers. The lower response rate impacts the generalizability of findings for fathers, specifically. Our response rate could have been improved by an earlier reminder, perhaps 2 weeks after the questionnaires were initially mailed out. Lastly, our sample did not represent the general population. Our sample was predominantly white with higher income and education levels. Therefore, additional research is required with a more diverse sample. We suggest additional studies be undertaken in this area to corroborate our findings. Future studies could employ an experimental method where families are randomized to receive different preparations before the transfer, different levels of support both during and after the transfer process. This additional information may help further identify areas for improvement in various transfer processes involving vulnerable infants.

## Conclusion

Our findings suggest that NICUs can implement strategies to help ease distress and dissatisfaction in parents with their neonates’ retro-transfer. Providing at least 24 hours’ notice, regular transfer updates, employing anticipatory preparation strategies, and fostering open communication between parents and health care professionals will help ensure parental satisfaction with the retro-transfer process.

## Data Availability

The datasets generated and/or analysed during the current study are not publicly available due the sensitive nature of the topic but are available from the corresponding author on reasonable request.
